# Evaluation of the Effects of Pecto-Intercostal Fascial Plane Blocks on Extubation Time in Cardiac Surgery: A Retrospective Study

**DOI:** 10.3390/jcm15114117

**Published:** 2026-05-26

**Authors:** Anıl Onur, Tuğba Onur, Ümran Karaca, Filiz Ata, Canan Yılmaz, Ayşe Neslihan Balkaya, Ahmet Burak Tatlı, Buket Özyaprak, Asiye Demirel, Nermin Kılıçarslan, Şeyda Efsun Özgünay, Osman Sıla Aydın, Cihan Sedat Aytünür, Füsun Gözen

**Affiliations:** 1 Department of Anesthesiology and Reanimation, University of Health Sciences, Bursa Yüksek İhtisas Training and Research Hospital, Bursa 16310, Turkey; doktor-t@hotmail.com (T.O.); umransuna@gmail.com (Ü.K.); filizatafiliz@hotmail.com (F.A.); dr_cnnylmz@yahoo.com (C.Y.); aynesbalkaya@gmail.com (A.N.B.); b.ozyaprak@hotmail.com (B.Ö.); dr.asiyedemirel@hotmail.com (A.D.); nerminkilicarslan2001@gmail.com (N.K.); seyda-efsun@hotmail.com (Ş.E.Ö.); osmansila@gmail.com (O.S.A.); dr.csa@msn.com (C.S.A.); fusungozen@gmail.com (F.G.); 2 Department of Cardiovascular Surgery, University of Health Sciences, Bursa Yüksek İhtisas Training and Research Hospital, Bursa 16310, Turkey; ahmetburaktatli@gmail.com

**Keywords:** pecto-intercostal fascial plane block, cardiac surgery, extubation time, opioid consumption, regional anesthesia, postoperative analgesia

## Abstract

**Background:** Prolonged extubation and pain following cardiac surgery remain significant clinical challenges. The pecto-intercostal fascial plane block (PIFB) is an emerging regional anesthesia technique incorporated into multimodal analgesia protocols to reduce opioid consumption and facilitate early extubation. This study retrospectively evaluated extubation times, perioperative opioid consumption, and postoperative analgesic requirements in patients who underwent isolated open-heart surgery via median sternotomy, comparing those who received PIFB with those who did not. **Methods:** This retrospective single-center study included ninety-nine patients who underwent isolated on-pump coronary artery bypass graft surgery via median sternotomy between 1 June 2023 and 25 March 2024. The study included 46 patients who received PIFB (Group 1) and 53 patients who received no block (Group 2). Ultrasound-guided bilateral PIFB was performed after anesthesia induction, with a total of 40 mL administered to each side (30 mL 0.25% bupivacaine + 10 mL normal saline). Demographic data, perioperative data, extubation times, analgesic consumption, and complications were compared between groups. **Results:** Demographic data, EuroSCORE, body mass index, and ejection fraction were similar between groups. Perioperative opioid (fentanyl) consumption was statistically significantly higher in Group 2 (median 450 [IQR: 350–600] μg vs. 400 [IQR: 350–450] μg; *p* = 0.037). Extubation time was statistically significantly shorter in Group 1 compared to Group 2 (median 340 [IQR: 265–490] min vs. 495 [IQR: 420–555] min; *p* < 0.001). The number of patients requiring postoperative paracetamol and tramadol was statistically significantly lower in Group 1 (*p* = 0.015 and *p* < 0.001, respectively). No statistically significant difference was found between groups regarding chest drain removal, length of hospital stay, or ICU length of stay (*p* > 0.05). Mortality occurred in 1 patient in Group 1 and 2 patients in Group 2. **Conclusions:** PIFB application in isolated open-heart surgery performed via median sternotomy was associated with shorter extubation time and reduced perioperative fentanyl and postoperative analgesic consumption, without a statistically significant effect on hospital length of stay. Complication and mortality data are reported descriptively; the study does not have sufficient statistical power to draw inferences regarding safety outcomes.

## 1. Background

Prolonged extubation and pain following cardiac surgery are significant clinical challenges. One of the subtitles of the Cardiac Enhanced Recovery After Surgery (ERAS) protocol is the reduction in opioid consumption through multimodal analgesia incorporating regional techniques for effective pain control after cardiac surgery [1,2,3]. Although several regional methods, such as epidural anesthesia and paravertebral block, are preferred in pain management after cardiac surgery, these techniques may raise concerns, particularly due to the risk of perioperative hypotension and potential hematoma [4,5]. In some studies, opioid use is still preferred as the primary method; furthermore, intravenous opioids are thought to allow effect titration with a higher safety profile intraoperatively compared to other routes of administration [6].

Publications suggesting that severe postoperative pain and increased opioid consumption in open-heart surgery performed via median sternotomy may be associated with prolonged extubation time, extended ICU and hospital stay, dysphagia, increased risk of postoperative pulmonary complications, and high hospital costs have led to the emergence of the pecto-intercostal fascial block (PIFB), which has been found effective in non-cardiac surgery, as a means of achieving effective pain control [7,8]. Some studies have reported that PIFB applied before anesthesia induction is associated with reduced intraoperative opioid consumption and shortened extubation time [9].

In some studies, the most significant factors affecting the length of ICU stay in patients undergoing coronary artery bypass graft (CABG) surgery were identified as intubation duration, body mass index (BMI), age, operative time, and the number of postoperative red blood cell transfusions [10]. Despite non-modifiable risk factors for prolonged extubation after cardiac surgery (advanced age, high New York Heart Association class, low ejection fraction), it is possible to investigate alternatives aimed at reducing perioperative opioid and anesthetic agent doses, preventing postoperative pain, and enabling early extubation as an anesthesia strategy. This study aimed to retrospectively evaluate extubation times, perioperative opioid consumption, length of hospital stay, and postoperative analgesic consumption in patients who underwent isolated open-heart surgery via median sternotomy at our institution, comparing those who routinely received PIFB with those who did not.

## 2. Methods

### 2.1. Study Design and Ethics

In accordance with decision number 2024/TBEK 2024/04-08, decision date 3 April 2024 from the Medical Sciences Research Ethics Committee of the University of Health Sciences, Bursa Yüksek İhtisas Training and Research Hospital, patients who underwent isolated coronary artery bypass graft surgery via median sternotomy in fully equipped cardiac surgery operating rooms between 1 June 2023 and 25 March 2024 were included in the study. The records of eligible patients were reviewed by a blinded anesthesiologist from hospital archives and records. This is a retrospective, single-center, observational cohort study using non-randomized convenience sampling. Group allocation was determined by routine clinical practice during the study period, as PIFB became part of institutional standard practice at our center during this time, resulting in unequal group sizes.

### 2.2. Patient Selection and Group Assignment

**Figure 1:**
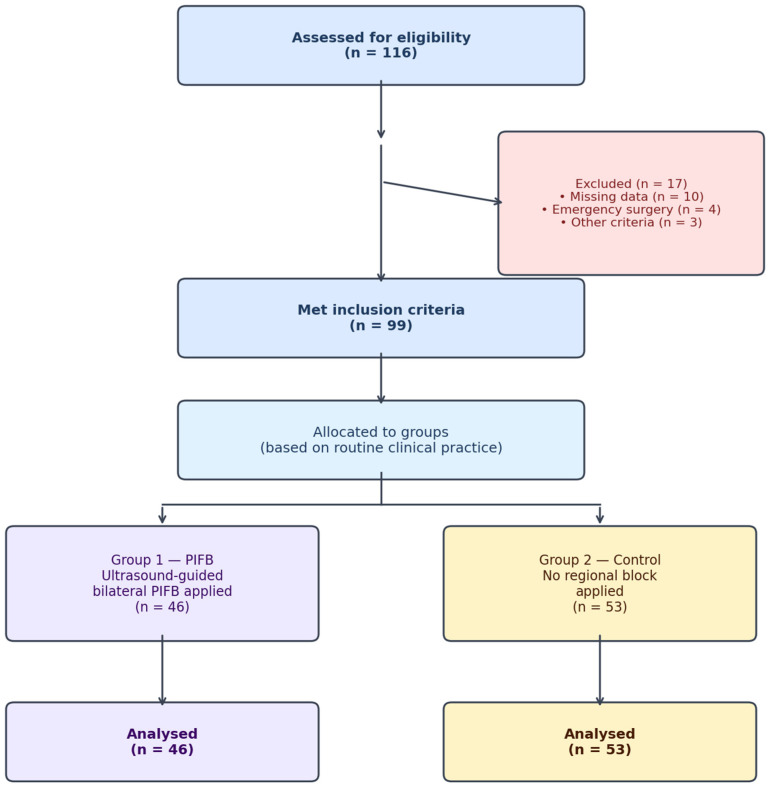
Patient flow diagram. Of 116 patients assessed for eligibility, 17 were excluded (10 missing data, 4 emergency surgery, 3 other criteria). The remaining 99 patients were allocated to Group 1 (PIFB, n = 46) and Group 2 (control, n = 53) based on routine clinical practice.

Patients aged 18–80 years who underwent elective open-heart surgery via median sternotomy during the specified period were included in the study (the 18–80 age range was applied as a standard institutional criterion). All patients underwent preoperative pulmonary function testing (PFT); patients with advanced chronic obstructive pulmonary disease (COPD) or other serious pulmonary disease were excluded to ensure comparable baseline pulmonary health status between groups. Exclusion criteria were: missing file information; inability to be contacted; non-Turkish speaking; prior history of cerebrovascular disease or cognitive impairment; advanced COPD or serious pulmonary disease confirmed by preoperative PFT; preoperative ejection fraction below 30%; renal insufficiency (requiring dialysis or serum creatinine > 2.0 mg/dL); known allergy to local anesthetics; emergency surgery; and concomitant valve or carotid surgery. These exclusions were applied to minimize confounding from comorbidities independently associated with prolonged mechanical ventilation and altered pharmacokinetics. Patients whose archival records were accessible were assigned to Group 1 (PIFB applied) and Group 2 (PIFB not applied). Of 116 patients initially assessed for eligibility, 17 were excluded: 10 due to missing file data, 4 due to emergency surgery, and 3 due to other exclusion criteria. The remaining 99 patients were included in the final analysis (see Figure 1).

### 2.3. Anesthesia Management

The routine anesthesia induction protocol consisted of standard monitoring in the operating room, radial artery catheterization, and anesthesia induction with midazolam 0.02–0.03 mg/kg intravenous (IV), propofol 2 mg/kg, fentanyl 2 μg/kg, and rocuronium 0.6 mg/kg IV, followed by intubation with an appropriately sized tube. Mechanical ventilation was set for all patients with FiO_2_: 50%, tidal volume: 6 mL/kg, positive end-expiratory pressure: 5 cm H_2_O, and respiratory rate: 12 breaths/min. Sevoflurane was administered at 0.8–1 minimum alveolar concentration for anesthesia maintenance. Fentanyl was the sole opioid used both for induction and intraoperative maintenance. Additional fentanyl boluses were administered by the attending anesthesiologist based on hemodynamic responses, targeting a mean arterial pressure of 70–80 mmHg. No other opioids were administered intraoperatively. Non-opioid analgesics were not routinely used intraoperatively in either group. Bispectral index (BIS) monitoring was not routinely performed during the study period.

### 2.4. Ultrasound-Guided PIFB Technique

Ultrasound-guided PIFB is a regional anesthesia and analgesia technique targeting the anterior branches of the intercostal nerves innervating the anterior chest wall. In our clinic, PIFB is routinely applied bilaterally under ultrasound guidance in the supine position after anesthesia induction. A linear ultrasound probe (GE Healthcare Logiq P5, GE HealthCare Technologies, Inc., Chicago, IL, USA) is placed 2–3 cm lateral to the sternal edge at the level of the 4th and 5th ribs, visualizing the pectoralis major muscle, intercostal muscle, intercostal thoracic vessels, and transversus thoracis muscle. Using an in-plane technique, a 21-gauge needle is placed between the pectoralis major muscle and the intercostal interfascial plane at the 4th intercostal space, and local anesthetic is administered. After confirming needle placement by hydrodissection and observing muscle separation, a fixed total volume of 40 mL (30 mL 0.25% bupivacaine + 10 mL normal saline) was injected bilaterally, regardless of patient weight.

### 2.5. Postoperative ICU Management and Extubation Criteria

All patients were transferred to the cardiovascular surgery intensive care unit (ICU) in the postoperative period and followed by the same team throughout the study period. Mechanical ventilation was maintained to ensure adequate oxygenation and ventilation. Sedation was gradually reduced in accordance with standard clinical practice; extubation was evaluated equally for all patients regardless of group assignment when the following criteria were met: mean arterial pressure > 65 mmHg without escalating vasopressor support; respiratory rate 10–30 breaths/min with tidal volume ≥ 5 mL/kg on minimal ventilatory support; adequate neuromuscular recovery (train-of-four ratio ≥ 0.9 where applicable); chest tube output < 200 mL/h; ability to follow simple commands (GCS motor score ≥ 6); and core temperature ≥ 36 °C. Postoperative pain management was conducted by the cardiovascular surgery ICU team under a standardized stepwise institutional protocol. All patients routinely received intravenous paracetamol (3 × 1 g/day) as first-line analgesia. In cases of inadequate pain control, as determined by the attending ICU clinician based on clinical assessment, tramadol (3 × 1 amp/day) was added. No postoperative opioids were administered. Formal VAS/NRS pain scoring was not part of routine ICU documentation during the study period; the decision to administer rescue analgesia was therefore based on the clinician bedside assessment rather than a predefined numerical pain threshold.

### 2.6. Data Collection

Demographic characteristics (age, sex, body mass index, American Society of Anesthesiologists [ASA] values, comorbidities, medications, EuroSCORE, ejection fraction) of patients who underwent elective isolated open-heart surgery via median sternotomy were recorded from patient files. Whether PIFB was applied before anesthesia induction and local anesthetic doses, perioperative complications, pump and cross-clamp times, operative time, opioid doses used, total analgesic dose within 24 h, time of first analgesic use, extubation time, discharge, ICU length of stay, time of chest drain removal, mortality (occurring within 10 postoperative days), complications, need for reintubation, and delirium scales used routinely were obtained from patient records.

### 2.7. Endpoints

The primary endpoint was to determine extubation times and total perioperative opioid consumption in patients who underwent surgery during the specified dates. Secondary endpoints were total analgesic doses used in the first 24 h, drain removal time, ICU and hospital length of stay, complications, opioid side effects, and mortality.

### 2.8. Statistical Analysis

Descriptive statistics were calculated for all variables. The distribution of continuous variables was assessed using the Shapiro–Wilk test and histogram plots. Normally distributed continuous variables are presented as mean ± standard deviation (SD), while non-normally distributed continuous variables are presented as median and interquartile range (IQR). Categorical variables are expressed as frequencies. For comparisons of continuous variables between groups, Student’s *t*-test was used for normally distributed data and the Mann–Whitney U test for non-normally distributed data. Pearson chi-square or Fisher’s exact test was applied as appropriate for categorical variables. Linear regression analysis was performed to identify factors associated with extubation time. Variables considered to have a potential relationship with the dependent variable were first evaluated in univariate analyses; those found to be significant were included in a multivariate model using the backward elimination method. Multivariate logistic regression analysis was performed for categorical dependent variables; results were reported as odds ratios (OR) with 95% confidence intervals (CI). As a post hoc power analysis, effect size (r) was derived from the Mann–Whitney U test Z score (r = |Z|/√N), yielding r = 0.37, which was converted to Cohen’s d = 0.80. Based on this effect size, the statistical power of the study was calculated as 97.6% using G*Power 3.1.9.7. Statistical analyses were performed with SPSS version 26.0 (IBM Corp., Armonk, NY, USA). A *p* value of <0.05 was considered statistically significant.

## 3. Results

A total of 99 patients met the eligibility criteria and were included in the final analysis. Of these, 46 formed Group 1 (PIFB applied) and 53 formed Group 2 (no regional block applied). The patient flow diagram is presented in Figure 1. Details of group allocation and study design are described in Section 2.1 and Section 2.2.

No statistically significant difference was found between groups regarding demographic data (*p* > 0.05) (Table 1). Patients in both groups were predominantly male and ASA III. EuroSCORE, body mass index, and ejection fraction were found to be similar. Normality of continuous demographic variables was confirmed using the Shapiro–Wilk test; accordingly, these variables are presented as mean ± standard deviation (SD) in Table 1, and group comparisons were performed using Student’s *t*-test.

Anesthesia and operative times and perioperative opioid consumption were statistically significantly higher in Group 2 (*p* = 0.011, *p* = 0.007, and *p* = 0.004, respectively). Cross-clamp and pump times were statistically similar between the two groups (*p* > 0.05) (Table 2).

Regarding extubation time, Group 1 was found to be statistically significantly shorter compared to Group 2 (*p* < 0.001) (Table 3). When paracetamol and tramadol doses used for postoperative analgesia were evaluated, Group 1 was found to be statistically significantly lower (*p* = 0.015 and *p* < 0.001, respectively) (Table 3).

Postoperative complications and mortality data were recorded descriptively for both groups. Complications were observed in 11 patients in Group 1 (7 bleedings resolved without reoperation, 1 delirium, 1 revision surgery, 1 transient cerebrovascular event, 1 renal complication) and in 9 patients in Group 2 (6 delirium, 2 bleedings not requiring reoperation, 1 renal complication). Reintubation was required in 2 patients in Group 1 and in no patients in Group 2. These data are presented for descriptive purposes only; since the study does not have sufficient statistical power to detect differences in complication rates or mortality, no statistical comparison between groups was performed for these outcomes, and no safety inferences should be drawn from these data. When assessed in terms of additional analgesic requirement within the first 24 h, analgesia was required in 6 patients in Group 1 and 47 patients in Group 2.

No statistically significant difference was found between groups regarding thoracic drain removal time, hospital stay, and ICU length of stay (*p* > 0.05) (Table 3). The mean thoracic drain removal time was 3.67 days in Group 1 and 3.01 days in Group 2.

Mortality within 10 postoperative days was recorded in 1 patient in Group 1 and 2 patients in Group 2; these data are presented descriptively only, and no comparative inference is made due to the insufficient statistical power for safety outcomes of the study.

In the univariate linear regression analysis performed to identify factors associated with extubation time, statistically significant associations were found between extubation time and block application and age. Block application was negatively associated with extubation time (B = −149.675; *p* = 0.019), indicating a shorter extubation time in patients who received the block. Similarly, age was positively associated with extubation time (B = 6.892; *p* = 0.022), with increasing age associated with prolonged extubation. BMI (*p* = 0.916), EF (*p* = 0.197), ASA (*p* = 0.206), anesthesia duration (*p* = 0.122), and operative time (*p* = 0.139) were not significantly associated with extubation time. In the multivariate regression analysis using the backward elimination method, both age (B = 7.344; *p* = 0.012) and block application (B = −159.028; *p* = 0.011) remained as independent predictors of extubation time. The final model yielded R^2^ = 0.117, explaining approximately 11.7% of the variance in extubation time (Table 4).

In the multivariate logistic regression analysis evaluating factors associated with tramadol use, block application was identified as an independent and statistically significant predictor. Tramadol use was significantly lower in patients who received the block compared to those who did not (OR = 0.103; 95% CI: 0.034–0.315; *p* < 0.001). Age (*p* = 0.337), BMI (*p* = 0.279), EF (*p* = 0.294), EuroSCORE (*p* = 0.243), and ASA class (*p* = 0.101) were not significantly associated with tramadol use. Nagelkerke R^2^ = 0.427 (Table 5).

## 4. Discussion

In our study, patients who received PIFB demonstrated a significant reduction in perioperative opioid consumption, shortened extubation time, and decreased postoperative paracetamol and tramadol doses. Complication and mortality data are presented descriptively; since the study does not have sufficient statistical power to evaluate safety outcomes, no comparative inference is made for these endpoints.

The primary finding of this study is that PIFB was associated with a statistically significant reduction in perioperative fentanyl consumption and shorter extubation time compared to patients who did not receive the block. These results are consistent with the proposed analgesic mechanism of PIFB, which targets the anterior branches of the intercostal nerves innervating the anterior chest wall, thereby reducing nociceptive input from the sternotomy incision and potentially attenuating the hemodynamic stress response that increases intraoperative opioid requirements [11].

When evaluated in terms of postoperative analgesic consumption, the significantly lower rates of paracetamol and tramadol use in the PIFB group are consistent with an opioid-sparing effect extending into the postoperative period. A plausible explanation is that the residual analgesic effect of bupivacaine administered at induction continues to attenuate pain signaling during the early postoperative hours, thereby lowering the threshold for rescue analgesia.

In our clinic, PIFB is routinely applied after anesthesia induction or at sternal closure for pain management after cardiac surgery. Analysis of acute postoperative pain is important because adequate pain management during the first few days after surgery is largely dependent on the success of the surgical treatment. Postoperative pain originates from tissue damage caused during the surgical procedure [12]. The postoperative analgesia process was managed by the cardiovascular surgery team in accordance with a standardized stepwise protocol using multimodal anesthesia [13]: intravenous paracetamol was applied as first-line analgesia, and tramadol was used in cases of inadequate pain control. No postoperative opioids were used.

In the present study, fentanyl was used as the perioperative opioid, and all opioid-related findings pertain specifically to fentanyl. In contrast, some studies in the literature have used sufentanil as the primary opioid in cardiac surgery. In one study using sufentanil, bilateral PIFB was associated with reduced perioperative sufentanil dose, shorter mean extubation time, and shorter ICU stay; these benefits were attributed to the improved pain control achieved through PIFB [9].

Fentanyl is routinely preferred as the perioperative opioid in our clinic. In our study, although cross-clamp and pump times were similar in patients with and without block, perioperative fentanyl volume was lower in Group 1. Routine bispectral index monitoring cannot be performed during surgery; additional anesthetic agents and inhalation maintenance are administered at certain periods during the pump. Fentanyl is administered by anesthesiologists according to hemodynamic responses to maintain mean arterial pressure around 70–80 mmHg. We believe that the reduced postoperative analgesic requirement and shorter extubation time in patients who received the block may be related to lower fentanyl doses.

Although publications suggesting effective postoperative pain control and reduced complications with ultrasound-guided PIFB are increasing in cardiac surgery, there are differing views in the literature regarding the duration of mechanical ventilation. While some studies have varying interpretations of the effect of PIFB on extubation time, the majority of publications indicate that it is an easy and effective block in cardiac surgery that reduces cumulative opioid consumption [9,14,15,16]. A similar study demonstrated that patients who received ultrasound-guided parasternal block had a significant reduction in intraoperative opioid consumption, extubation time, and postoperative spirometry performance compared to the control group, with optimal perioperative analgesia achieved [17]. In our study, extubation time was observed to be shorter in patients who received the block compared to those who did not. However, spirometric evaluation records could not be accessed. Additionally, a study examining the timing of block administration reported similar pain outcomes between preoperative and postoperative parasternal intercostal block application [18]. In our study, PIFB was performed after anesthesia induction and before incision.

Early extubation time continues to be an important issue for many reasons. A study identified postoperative complications such as pneumonia, respiratory failure, high drainage, need for intra-aortic balloon pump (IABP), higher EuroSCORE 2, cardiac dysfunction, cerebrovascular event, and epinephrine use as independent risk factors for prolonged intubation after cardiac surgery [19]. Early extubation within 8 h after cardiac surgery is also associated with improved resource utilization [20]. Studies show that early extubation in patients receiving low-dose rapid opioid cardiac anesthesia protocols is as safe as conventional care. Understanding factors associated with delayed extubation is critically important for perioperative planning and resource management, and studies on this subject continue [20].

Among postoperative complications, the literature includes many conditions such as myocardial ischemia, stroke, renal failure, bleeding, reintubation, pneumonia, and arrhythmia; similar complications are also descriptively noted in our study [21,22].

## 5. Limitations

Limitations of this study include a small number of patients, absence of perioperative bispectral index monitoring, lack of postoperative pulmonary function test records, retrospective design, absence of long-term follow-up and chronic pain monitoring. Additionally, the non-randomized convenience sampling method introduces a risk of selection bias, as group allocation was determined by routine clinical practice rather than randomization. The absence of a formally documented, objective extubation protocol may have introduced variability in extubation decisions between clinicians. The potential for performance bias also exists, as the anesthesia team was aware of group allocation. Since this is a retrospective study, a formal a priori sample size calculation was not performed; this may limit the statistical power of the findings. Importantly, post hoc power analysis was performed for the primary endpoint of extubation time, and the study does not have sufficient power to detect statistically significant differences in complication rates or mortality. Accordingly, complication and mortality data are presented only as descriptive observations; no safety inferences should be drawn from these data. The absence of validated postoperative pain scores (VAS/NRS) that were not routinely documented in patient records during the study period makes it impossible to directly and quantitatively assess pain intensity at specific postoperative time points, constituting a conceptual limitation in evaluating the analgesic mechanism of PIFB. The low R^2^ value (0.117) of the multivariate regression model reflects the inherent complexity of extubation time as a clinical outcome and the likelihood of residual confounding from unmeasured variables not captured in the retrospective dataset. The perioperative differences between groups (anesthesia duration, operative time, and opioid consumption) represent potential confounders that were not fully controlled for in the primary analysis. Future prospective randomized controlled studies should confirm these results with predetermined sample size calculations, formal extubation protocols, and standardized pain scoring.

## 6. Conclusions

This retrospective single-center study found that ultrasound-guided bilateral PIFB was associated with shorter extubation time and reduced perioperative fentanyl and postoperative analgesic consumption in patients undergoing isolated on-pump CABG via median sternotomy, without adversely affecting hospital length of stay. Postoperative tramadol and paracetamol requirements were also significantly lower in the PIFB group, providing indirect evidence of an analgesic benefit extending into the postoperative period based on surrogate markers; direct pain assessment was not available in this retrospective dataset. These findings represent associations observed in an observational cohort and should not be interpreted as evidence of causation. The retrospective design, absence of formal pain scoring, non-randomized group allocation, and lack of a priori power calculation for secondary outcomes limit the strength of inferences that can be drawn. Nevertheless, this study provides real-world evidence from a high-volume cardiac surgery center and forms the rationale for prospective randomized controlled studies to definitively evaluate the role of PIFB in cardiac surgical analgesia, with standardized extubation protocols, validated pain assessment tools, and adequate sample sizes.

## Figures and Tables

**Table 1:** Demographic and preoperative characteristics of patients.

	Group 1 (n = 46)	Group 2 (n = 53)	*p*
F/M	14/32	13/40	0.510
ASA III/IV	43/3	45/8	0.176
BMI (kg/m^2^)	27.32 ± 3.12	27.41 ± 1.38	0.842
EuroSCORE	3.26 ± 1.52	3.11 ± 1.28	0.602
EF (%)	49.08 ± 6.55	48.01 ± 7.29	0.440

Data are presented as median (interquartile range, IQR) for continuous variables and number (n) for categorical variables. Continuous variables were compared using the Mann–Whitney U test and categorical variables using the chi-square test. *p* < 0.05 was considered statistically significant. ASA: American Society of Anesthesiologists; BMI: Body Mass Index; EF: Ejection fraction; F: Female; M: Male.

**Table 2:** Perioperative characteristics and outcomes.

	Group 1 (Mean ± SD)	Group 2 (Mean ± SD)	*p*
Anesthesia duration (min)	266.19 ± 53.42	293.58 ± 51.95	0.011 ᵃ
Operative time (min)	247.97 ± 48.58	275.84 ± 51.82	0.007 ᵃ
Pump time (min)	108 (88–118)	98 (80–126)	0.432 ᵇ
Cross-clamp time (min)	70.5 (56–88)	72 (55–88)	0.983 ᵇ
Total opioid volume (μg)	400 (350–450)	450 (350–600)	0.037 ᵇ

min: minutes. Data are presented as mean ± standard deviation (SD) for normally distributed variables and median (interquartile range, IQR) for non-normally distributed variables. Group comparisons were made using Student’s *t*-test ^a^ for normally distributed data and the Mann–Whitney U test ^b^ for non-normally distributed data.

**Table 3:** Postoperative outcomes and complications.

	Group 1 (n = 46)	Group 2 (n = 53)	*p*
Extubation time (min)	340 (265–490)	495 (420–555)	<0.001 **
Thoracic drainage time (days)	2 (2–4)	3 (3–3)	0.108
ICU length of stay (days)	3 (2–4)	3 (3–4)	0.089
Hospital discharge time (days)	7.5 (7–12)	8 (7–8)	0.852
Patients requiring paracetamol	14/32 ^†^	29/24 ^†^	0.015 *
Patients requiring tramadol	5/41 ^†^	28/25 ^†^	<0.001 **

Data are presented as median (interquartile range, IQR) for continuous variables and number (n) for categorical variables. Continuous variables were compared using the Mann–Whitney U test and categorical variables using the chi-square test. * *p* < 0.05; ** *p* < 0.001 was considered statistically significant. ICU: Intensive Care Unit. ^†^ Data are presented as number requiring/not requiring medication.

**Table 4:** Univariate and multivariate linear regression analysis of factors associated with extubation time.

Variable	Univariate B	Std. B	*p*	Multivariate B	Std. B	*p*
Block	−149.675	−0.237	0.019	−159.028	−0.252	0.011
Age (years)	6.892	0.231	0.022	7.344	0.246	0.012
BMI (kg/m^2^)	−1.450	−0.011	0.916	—	—	—
EF (%)	−6.038	−1.300	0.197	—	—	—
ASA	128.417	0.129	0.206	—	—	—
Anesthesia duration (min)	0.923	0.157	0.122	—	—	—
Operative time (min)	0.913	0.151	0.139	—	—	—

B: Unstandardized regression coefficient; Std. B: Standardized regression coefficient; EF: Ejection fraction; ASA: American Society of Anesthesiologists; BMI: Body Mass Index. Variables not included in the multivariate model are indicated with—Multivariate model R^2^ = 0.117.

**Table 5:** Multivariate logistic regression analysis of factors associated with tramadol use.

Variable	OR (Exp(B))	95% CI	*p*
Block	0.103	0.034–0.315	<0.001
Age (years)	1.021	0.979–1.065	0.337
BMI (kg/m^2^)	1.081	0.939–1.245	0.279
EF (%)	0.966	0.906–1.030	0.294
EuroSCORE	0.808	0.565–1.156	0.243
ASA	3.419	0.787–14.856	0.101

OR: Odds ratio; CI: Confidence interval; EF: Ejection fraction. Variables included in the model: Block, age, BMI, EF, EuroSCORE, and ASA class. Reference categories are ‘no block’ for Block and ‘ASA III’ for ASA class. Nagelkerke R^2^ = 0.427.

## Data Availability

The datasets used and/or analyzed during the current study are available from the corresponding author upon reasonable request.
